# Eight Collagen Peptides from Hydrolysate Fraction of Spanish Mackerel Skins: Isolation, Identification, and In Vitro Antioxidant Activity Evaluation

**DOI:** 10.3390/md17040224

**Published:** 2019-04-13

**Authors:** Jing-Bo Zhang, Yu-Qin Zhao, Yu-Mei Wang, Chang-Feng Chi, Bin Wang

**Affiliations:** 1 National and Provincial Joint Laboratory of Exploration and Utilization of Marine Aquatic Genetic Resources, National Engineering Research Center of Marine Facilities Aquaculture, School of Marine Science and Technology, Zhejiang Ocean University, 1st Haidanan Road, Zhoushan 316022, China; gbozhang1993@163.com; 2 Zhejiang Provincial Engineering Technology Research Center of Marine Biomedical Products, School of Food and Pharmacy, Zhejiang Ocean University, 1st Haidanan Road, Zhoushan 316022, China; zhaoy@hotmail.com (Y.-Q.Z.); wangym731@126.com (Y.-M.W.)

**Keywords:** spanish mackerel (*Scomberomorous niphonius*), skin, antioxidant activity, collagen hydrolysate, peptide

## Abstract

A previous report indicated that collagen hydrolysate fraction (F7) from Spanish mackerel (*Scomberomorous niphonius*) skins showed high reducing power and radical scavenging activities on 2,2-Diphenyl-1-picrylhydrazyl (DPPH) (EC_50_ value of 1.57 mg/mL) and hydroxyl (EC_50_ value of 1.20 mg/mL). In this work, eight peptides were isolated from F7 and identified as Gly-Pro-Tyr (GPY, 335.31 Da), Gly-Pro-Thr-Gly-Glu (GPTGE, 459.47 Da), Pro-Phe-Gly-Pro-Asp (PFGPD, 531.52 Da), Gly-Pro-Thr-Gly-Ala-Lys (GPTGAKG, 586.65 Da), Pro-Tyr-Gly-Ala-Lys-Gly (PYGAKG, 591.69 Da), Gly-Ala-Thr-Gly-Pro-Gln-Gly (GATGPQG, 586.61 Da), Gly-Pro-Phe-Gly-Pro-Met (GPFGPM, 604.73 Da), and Tyr-Gly-Pro-Met (YGPM, 466.50 Da), respectively. Among them, PFGPD, PYGAKG, and YGPM exhibited strong radical scavenging activities on DPPH (EC_50_ values of 0.80, 3.02, and 0.72 mg/mL for PFGPD, PYGAKG, and YGPM, respectively), hydroxyl (EC_50_ values of 0.81, 0.66, and 0.88 mg/mL for PFGPD, PYGAKG, and YGPM, respectively), superoxide anion (EC_50_ values of 0.91, 0.80, and 0.73 mg/mL for PFGPD, PYGAKG, and YGPM, respectively), and 2,2′-azino-bis(3-ethylbenzothiazoline-6-sulphonic acid) (ABTS) cation (EC_50_ values of 0.86, 1.07, and 0.82 mg/mL for PFGPD, PYGAKG, and YGPM, respectively) in a positive concentration–activity relationship. Furthermore, PFGPD, PYGAKG, and YGPM could effectively reduce Fe^3+^ to Fe^2+^ and inhibit lipid peroxidation. Hence, eight collagen peptides from hydrolysate of Spanish mackerel skins might be served as antioxidant candidates for various industrial applications.

## 1. Introduction

Reactive oxygen species (ROS) derived from molecular oxygen are indispensable cellular components in organisms that take part in cell signaling and homeostasis during physiological processes [1]. However, excess ROS during oxidative stress can result in oxidative damage to cell membranes, proteins, and DNA. Those damage will cause increased issues in a series of chronic diseases including hepatopathy, atherosclerosis, and diabetes [1,2]. In addition, radical mediated oxidation can cause deterioration in food products, which is a main concern during food processing and storage. This concern is because the toxic and smelly secondary metabolites produced by lipid peroxidation can negatively influence normal functions within the human body [3,4]. Therefore, chronic diseases and food spoilage caused by excess ROS arouse widespread public concern because they could result in great damage to public health and food industries. At present, chemically synthesized antioxidants have been generally applied to biomedical products, daily cosmetics, and foodstuff industries [5]. However, the general public have an increasing awareness of food safety and the potential toxicity of chemical preservatives and antioxidants, which has urged researchers to develop new approaches to exploit innoxiously natural antioxidants [6]. Antioxidant peptides (APs) derived from food waste can promote human health through decreasing the risk of chronic diseases related to oxidative stress. In addition, APs can effectively delay food deterioration caused by lipid peroxidation [2,7]. Therefore, APs have been proposed as a perfect alternative to chemical antioxidants due to their bioactivities, safety, and nutritional properties [2]. 

As reported by Food and Agriculture Organization of the United Nations (FAO) in 2016, the quantity of global fish production is about 1.71 × 10^8^ tons. Commercial fish processing generates a large amount of fish by-products, which cause heavy processing and environmental problems [2]. Therefore, collagens and peptides, with excellent physicochemical properties and antioxidant activity, have been prepared using seafood by-products including skin [8,9], bone [5,10], head [11,12], scale [6], and dark muscle [13]. Jridi et al. researched the structural and rheological properties and the enhanced wound-healing ability of collagen-based gel obtained from cuttlefish skin, and the results indicated that cuttlefish collagen-based gel might be useful as a wound healing agent [9]. Previous reports have indicated that collagen hydrolysate and peptides from cod skin can protect mice skin from ultraviolet radiation-induced damage including wrinkle formation and destruction, through suppressing the depletion of endogenous antioxidant enzyme and inhibiting the expression of NF-κB and pro-inflammatory cytokines mediated by NF-κB [14]. In addition, the peptides have shown high moisture retention and absorption abilities. Chi et al. [3,12] reported that APs including GVPLT, GPGGFI, FIGP, WEGPK, GPP, and GSGGL from protein hydrolysate of bluefin leatherjacket head and skin exhibited high scavenging activities on superoxide anion radical ($O_{2}^{-}$•), hydroxyl radical (HO•), and 2,2-Diphenyl-1-picrylhydrazyl radical (DPPH•). WEGPK and FIGP could effectively inhibit the peroxidation of unsaturated fatty acids. Their antioxidant activities are related to their molecular structures, such as small molecular sizes, amino acid (AA) sequences, and the ratios of hydrophobic and aromatic AAs [6]. In addition, collagens and APs from marine organisms have also shown strong activity and potential application value [15]. Pozzolini et al. reported that collagen-derived peptides from *Chondrosia reniformis* might be used in drug and cosmetic formulations for damaged or photoaged skin repair because it could stimulate and increase cell growth and proliferation, scavenging ROS, and protect cells from UV-induced death [16]. Zhao et al. reported that peptide fraction (SBP-III-3) from croceine croaker swim bladder possessed good antioxidant and anti-fatigue capacities on mice by protecting DNA from oxidative damage and increasing the activities of superoxide dismutase, catalase, and glutathione peroxidase [17].

Spanish mackerel (*Scomberomorous niphonius*) is an important aquaculture species in China. During factory processing, an enormous amount of skins of this species are generated as a by-product [18]. To take full advantage of Spanish mackerel, we successfully prepared its skin collagens, collagen hydrolysate, and hydrolysate fractions (F1 to F7) with average molecular weight (AMW) ranging from 5.04 to 47.82 kDa [19]. Furthermore, the relationships between the physicochemical properties and AMW of seven hydrolysate fractions (F1 to F7), and hydrolysate fraction of F7 with minimum AMW (5.04 kDa) showed the highest reducing power and radical scavenging activities on DPPH• (EC_50_ value of 1.57 mg/mL) and HO• (EC_50_ value of 1.20 mg/mL), among all hydrolysate fractions [19]. Therefore, the aim of the study was focused on the preparation, identification, and activity evaluation of antioxidant peptides from hydrolysate fraction (F7) of Spanish mackerel. 

## 2. Results and Discussion

### 2.1. Purification of APs from Collagen Hydrolysate Fraction of F7

#### 2.1.1. Gel Filtration Chromatography of F7 Using Superdex^®^ Peptide 10/300 GL Column

**Figure 1:**
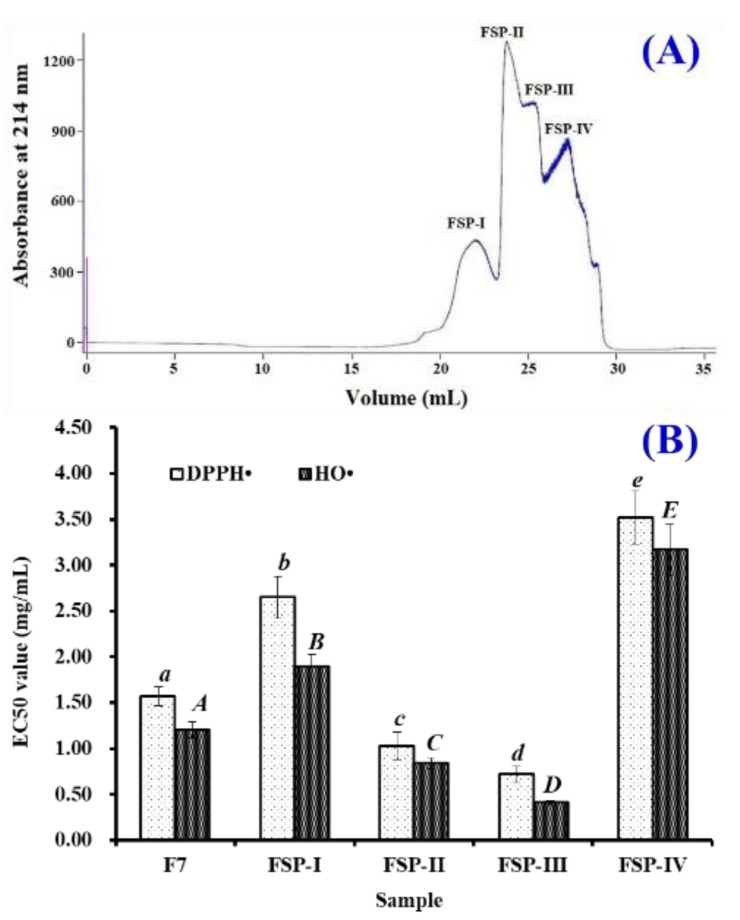
Elution profile (**A**) and EC_50_ values on DPPH• and HO• (**B**) of four fractions (FSP-I to FSP-IV) from F7 by ÄKTA avant 25 with a Superdex^®^ Peptide 10/300 GL column. All data are presented as the mean ± SD of triplicate results. ^a–e/A–E^ Values with same superscripts of this type indicate no significant difference (*p* > 0.05).

Superdex^®^ Peptide 10/300 GL is designed for high-efficiency and lab-scale isolation of biomolecules including peptides and proteins with molecular weights (MWs) between 0.1 and 7.0 kDa. Therefore, hydrolysate fraction of F7 was further separated using the column and divided into four components, defined as FSP-I, FSP-II, FSP-III, and FSP-IV, respectively (Figure 1A). Their DPPH• and HO• scavenging activities are shown in Figure 1B. These data indicated that EC_50_ values of FSP-III on DPPH• and HO• were 0.72 ± 0.09 and 0.41 ± 0.02 mg/mL, which were significantly lower than those of F7 (DPPH•: 1.57 ± 0.12 mg/mL; HO•: 1.20 ± 0.09 mg/mL), FSP-I (DPPH•: 2.65 ± 0.23 mg/mL; HO•: 1.89 ± 0.14 mg/mL), FSP-II (DPPH•: 1.03 ± 0.15 mg/mL; HO•: 0.84 ± 0.06 mg/mL), and FSP-IV (DPPH•: 3.52 ± 0.29 mg/mL; HO•: 3.17 ± 0.28 mg/mL), respectively (*p* < 0.05). Therefore, FSP-III should contain more APs amount and was suitable for the following separation process.

#### 2.1.2. Isolation of APs from FSP-III by RP-HPLC

**Figure 2:**
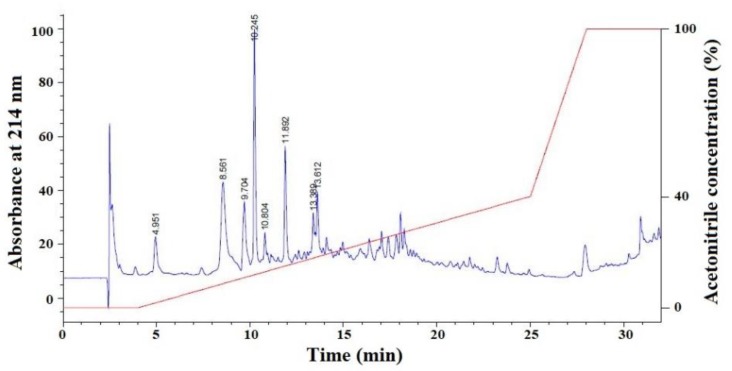
Elution profile of FSP-III separated on a Kromasil C18 column (10 × 250 mm) in RP-HPLC system.

FSP-III with high radical scavenging activities was further purified on a Kromasil C18 column (10 × 250 mm) in RP-HPLC system and the result is presented in Figure 2. According to the chromatographic peaks, eight fractions with retention time (RT) of 4.951 min (F7-P1), 8.561 min (F7-P2), 9.704 min (F7-P3), 10.245 min (F7-P4), 10.804 min (F7-P5), 11.892 min (F7-P6), 13.389 min (F7-P7), and 13.612 min (F7-P8), respectively, were purified and enriched through repeated chromatography for the identification of AA sequences.

### 2.2. Determination of AA Sequence

The AA sequences of eight isolated peptides (F7-P1 to F7-P8) were analyzed using a Protein/Peptide Sequencer (Table 1), and their sequences were determined to be Gly-Pro-Tyr (GPY, F7-P1), Gly-Pro-Thr-Gly-Glu (GPTGE, F7-P2), Pro-Phe-Gly-Pro-Asp (PFGPD, F7-P3), Gly-Pro-Thr-Gly-Ala-Lys (GPTGAKG, F7-P4), Pro-Tyr-Gly-Ala-Lys-Gly (PYGAKG, F7-P5), Gly-Ala-Thr-Gly-Pro-Gln-Gly (GATGPQG, F7-P6), Gly-Pro-Phe-Gly-Pro-Met (GPFGPM, F7-P7), and Tyr-Gly-Pro-Met (YGPM, F7-P8), respectively. Using electrospray ionization-mass spectrometer (ESI-MS), molecular masses of eight isolated peptides (F7-P1 to F7-P) were determined as 335.31 Da, 459.47 Da, 531.52 Da, 586.65 Da, 591.69 Da, 586.61, 604.73 Da, and 466.50 Da, respectively, which agreed well with their theoretical masses of 335.36, 459.45 Da, 531.56 Da, 586.64 Da, 591.66 Da, 586.60 Da, 604.72 Da, and 466.55 Da, respectively.

### 2.3. Antioxidant Activity

Radical scavenging assay, reducing power assay, and lipid peroxidation inhibition assay were employed to evaluate the activities of eight collagen peptides (F7-P1 to F7-P8), and their EC_50_ values on DPPH/hydroxyl/superoxide anion/ABTS radicals are presented in Table 2.

#### 2.3.1. Radical Scavenging Activity

##### DPPH• Scavenging Activity

**Figure 3:**
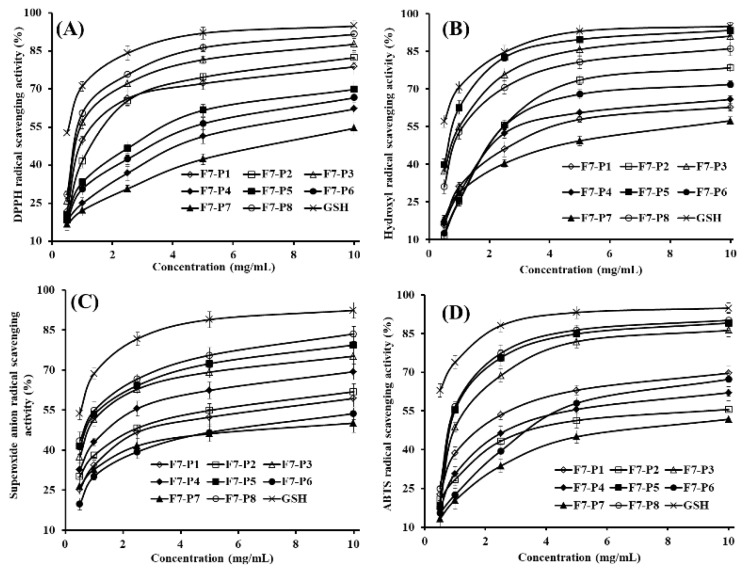
DPPH• (**A**), HO• (**B**), $O_{2}^{-}$• (**C**), and 2′-azino-bis(3-ethylbenzothiazoline-6-sulphonic acid) cation radical (ABTS^+^•) (**D**) scavenging activities of eight isolated peptides (F7-P1~F7-P8) from collagen hydrolysate fraction (F7) of Spanish mackerel (*S. niphonius*) skin. All data are presented as the mean ± SD of triplicate results.

DPPH, when reacted with an antioxidant, can generate the hydrazine DPPH, and the purple DPPH solution, with the maximum absorption wavelength 515 to 528 nm, will change to yellow [20,21]. As shown in Figure 3A, eight isolated collagen peptides (F7-P1 to F7-P8) scavenged DPPH• in a concentration-dependent manner, but they showed lower activity than the positive control (glutathione (GSH)). The EC_50_ values of F7-P3 and F7-P8 were 0.80 ± 0.09 mg/mL and 0.72 ± 0.06 mg/mL, respectively, which were significantly lower than the EC_50_ values of other six collagen peptides (*p* < 0.05). Moreover, EC_50_ values of F7-P3 and F7-P8 were significantly less than those of peptides from blue mussel (PYSFK: 1.575 mg/mL; YPPAK: 2.62 mg/mL; FLNEFLHV: 4.950 mg/mL) [7,22], bluefin leatherjacket heads (GPP: 1.927 mg/mL; GVPLT: 4.541 mg/mL; WEGPK: 4.438 mg/mL) [11], salmon pectoral fin (TTANIEDRR: 2.503 mg/mL) [23], loach (PSYV: 17.0 mg/mL) [24], skins of grass carp (GFGPL: 2.249 mg/mL; VGGRP: 2.937 mg/mL) [25], swim bladders of miiuy croaker (FTGMD: 2.22 mg/mL; YLPYA: 3.63 mg/mL; GFYAA: 5.02 mg/mL; FSGLR: 4.01 mg/mL; VPDDD: 2.87 mg/mL) [1], and cartilage of *Raja porosa* (GPAGDY: 3.48 mg/mL; IVAGPQ: 3.93 mg/mL, FIMGPY: 2.60 mg/mL) [5]. Those results indicated that eight isolated collagen peptides (F7-P1 to F7-P8) from Spanish mackerel skins, especially F7-P3 and F7-P8 can work as hydrogen donors and radical scavengers to inhibit the DPPH• chain reaction.

##### HO• Scavenging Activity

As the most ROS, HO• is involved in the induction of lipid peroxidation in the biological membrane, and it also has the ability to initiate carcinogenesis, mutagenesis, and cytotoxicity due to its effective reaction with biomacromolecules [7]. Figure 3B shows that eight isolated collagen peptides (F7-P1 to F7-P8) could effectively clear HO• in a dose–response relationship, and the HO• scavenging activities of F7-P3, F7-P5, and F7-P8 were the significantly better that those of the other six collagen peptides (*p* < 0.05), but weaker than that of GSH at the tested concentrations. EC_50_ values of F7-P3, F7-P5, and F7-P8 were 0.81 ± 0.05, 0.66 ± 0.08, and 0.88 ± 0.09 mg/mL, respectively. Those data were significantly lower than those of the other five collagen peptides (*p* < 0.05) (Table 2). Furthermore, EC_50_ values F7-P3, F7-P5, and F7-P8 were less than those of peptides from bluefin leatherjacket heads (WEGPK: 5.567 mg/mL; GPP: 2.385 mg/mL; GVPLT: 4.149 mg/mL) [12], weatherfish loach (PSYV: 2.64 mg/mL) [24], skins of grass carp (PYSFK: 2.283 mg/mL; GFGPL: 1.612 mg/mL; VGGRP: 2.055 mg/mL) [25], swim bladders of miiuy croaker (FYKWP: 1.45 mg/mL; FTGMD: 2.31 mg/mL; YLPYA: 2.90 mg/mL; GFYAA: 2.35 mg/mL; FSGLR: 2.45 mg/mL; VPDDD: 2.85 mg/mL) [1], and cartilage of *Raja porosa* (FIMGPY: 3.04 mg/mL; GPAGDY: 3.92 mg/mL; IVAGPQ: 5.03 mg/mL) [5]. Those data showed that eight isolated collagen peptides (F7-P1 to F7-P8) from Spanish mackerel skin could effectively scavenge HO• to lessen or eliminate injury caused by HO• in organisms.

##### $O_{2}^{-}$• Scavenging Activity

$O_{2}^{-}$• is a main cause of oxidative stress because it can facilitate oxidative reaction to bring forth hydrogen peroxide and HO• to attack susceptible biological targets including lipids, proteins, and nucleic acids, and alter their physiological function [13]. The data in Figure 3C indicated eight isolated collagen peptides (F7-P1 to F7-P8) that could positively scavenge $O_{2}^{-}$• in a dose–effect relation. As presented in Table 2, EC_50_ values of F7-P3 (0.91 ± 0.08 mg/mL), F7-P5 (0.80 ± 0.06 mg/mL), and F7-P8 (0.73 ± 0.06 mg/mL) were less than 1.0 mg/mL. Those data were significantly lower than the other six isolated collagen peptides (*p* < 0.05). However, the $O_{2}^{-}$• scavenging activity of eight isolated collagen peptides (F7-P1 to F7-P8) were lower than that of GSH at the tested concentrations. EC_50_ values of F7-P3 (0.91 ± 0.08 mg/mL), F7-P5 (0.80 ± 0.06 mg/mL), and F7-P8 (0.73 ± 0.06 mg/mL) were lower than those of APs from croceine croaker muscle (MILMR: 0.993 mg/mL) [3], cartilage of *Raja porosa* (GPAGDY: 1.66 mg/mL; FIMGPY: 1.61 mg/mL; IVAGPQ: 1.82 mg/mL) [5], bluefin leatherjacket heads (WEGPK: 3.223 mg/mL; GPP: 4.668 mg/mL; GVPLT: 2.881 mg/mL) [12], and swim bladders of miiuy croaker (FYKWP: 1.92 mg/mL; FTGMD: 3.04 mg/mL; YLPYA: 3.61 mg/mL; GFYAA: 3.03 mg/mL; FSGLR: 3.35 mg/mL; VPDDD:4.11 mg/mL) [1]. In living organisms, superoxide dismutase (SOD) protects the cell from the deleterious effects of superoxides. Therefore, eight isolated collagen peptides (F7-P1 to F7-P8), especially F7-P3, F7-P5, and F7-P8 might be used as the $O_{2}^{-}$• scavenger to help SOD to reduce $O_{2}^{-}$• damage in cells.

##### ABTS^+^• Scavenging Activity

ABTS^+^• scavenging assay is widely applied to detect the anti-radical ability of peptides [2]. In the measurement process, ABTS is oxidized by potassium persulfate to generate the blue/green ABTS^+^• with an absorption maximum of 734 nm. Furthermore, antioxidants can revert the blue/green color of ABTS^+^• solution back to its colorless neutral form, which follows the reduction of absorption at 734 nm [5,12]. As shown in Figure 3D, eight isolated collagen peptides (F7-P1 to F7-P8) exhibited powerful scavenging capability on ABTS^+^• in a dose–effect fashion. F7-P3 and F7-P8 with EC_50_ values of 0.86 ± 0.05 mg/mL and 0.82 ± 0.04 mg/mL had the most powerful scavenging capacity on ABTS^+^• among eight isolated collagen peptides (Table 2), but they were still weaker than the positive control of GSH at the tested concentrations. EC_50_ values of F7-P3 and F7-P8 were significantly less than those of APs from protein hydrolysates of food resources, including salmon muscle (FLNEFLHV: 1.548 mg/mL) [26], corn gluten meal (LLPF: 1.031 mg/mL; LPF: 1.013 mg/mL; FLPF: 1.497 mg/mL) [27], cartilage of *Raja porosa* (FIMGPY: 1.04 mg/mL; IVAGPQ: 1.29 mg/mL) [5], and bluefin leatherjacket heads (GVPLT: 3.124 mg/mL; GPP: 2.472 mg/mL; WEGPK: 5.407 mg/mL) [12]. These findings indicate that eight isolated collagen peptides (F7-P1 to F7-P8), especially F7-P3 and F7-P8 possess a high capacity to turn ABTS^+^• into its neutral form and inhibit free radical chain reactions.

#### 2.3.2. Reducing Power

**Figure 4:**
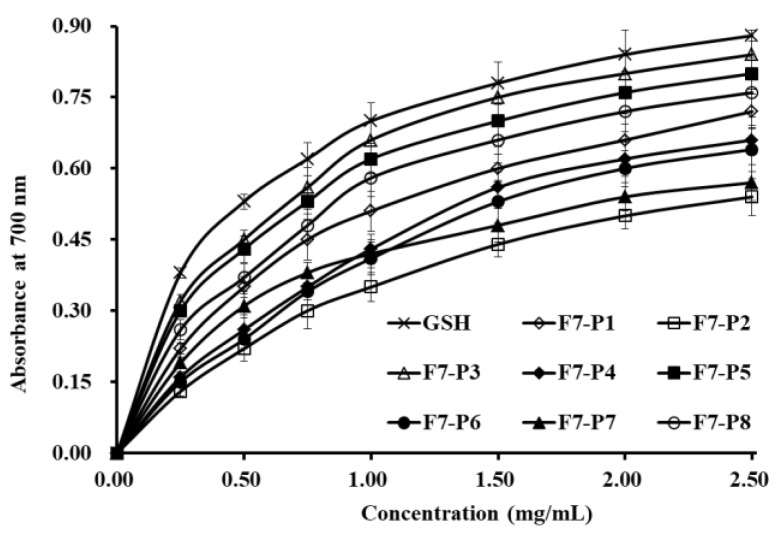
Reducing power of eight antioxidant peptides (F7-P1 to F7-P8) from collagen hydrolysate fraction (F7) of Spanish mackerel (*S. niphonius*) skin. All data are presented as the mean ± SD of triplicate results.

Reducing power is an important index applied to evaluate the antioxidant activities of bioactive peptides through the decrease of Fe ^3+^ to Fe ^2+^ [4]. As shown in Figure 4, eight isolated collagen peptides (F7-P1 to F7-P8) showed dose-dependent reducing power when the concentration of tested peptides increased from 0 to 2.5 mg/mL. The present data indicated that F7-P3 had the highest ability to decrease ferric ions (Fe^3+^) to ferrous ions (Fe^2+^) when compared with the other seven isolated collagen peptides, but still lower than GSH at the same tested concentrations.

#### 2.3.3. Lipid Peroxidation Inhibition Assay

**Figure 5:**
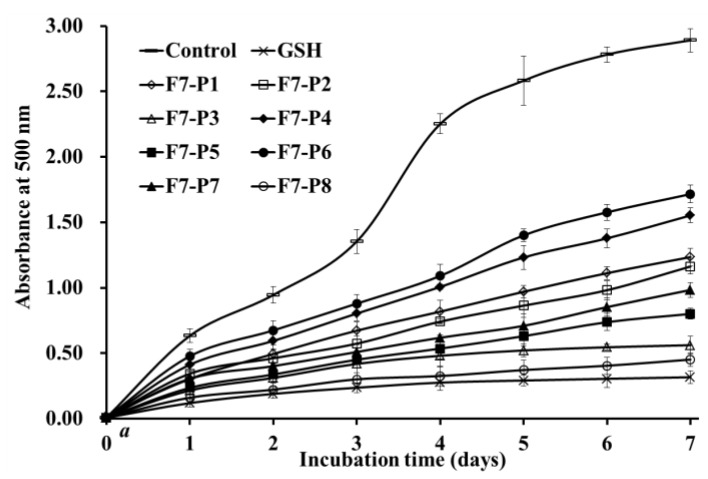
Lipid peroxidation inhibition ability of eight antioxidant peptides (F7-P1 to F7-P8) from collagen hydrolysate fraction (F7) of Spanish mackerel (*S. niphonius*) skin. All data are presented as the mean ± SD of triplicate results.

Polyunsaturated lipids in biological membranes are easily oxidized by ROS to produce alkanes, MDA, and 4-hydroxyl 2-nonenal. The process will lead to the lipid peroxidation [28]. Furthermore, secondary metabolites of lipid peroxidation can react with cell macromolecules to create deleterious adducts, which will cause serious irreversible consequences on the biological function of cells, such as membrane permeability and gene mutation [4,29]. As a consequence, capabilities of eight isolated collagen peptides (F7-P1 to F7-P8) on lipid peroxidation inhibition were investigated using the linoleic acid system [16,30]. In the system, detected sample solution with a high absorbance value at 500 nm indicates high oxidation degree of linoleic acid, which highlights that the sample has low capability on inhibiting the oxidation of unsaturated fatty acid. In comparison with the negative control group (without antioxidant), eight isolated collagen peptides (F7-P1 to F7-P8) could effectively inhibit the lipid peroxidation during 7 days at 40 °C (Figure 5). More importantly, the inhibiting ability of F7-P8 was close to that of the positive control of GSH and noticeably better than those of the other seven isolated collagen peptides. These data suggested that eight isolated collagen peptides (F7-P1 to F7-P8) could react with peroxyl radicals and inhibit the diffusion process of lipid peroxidation. 

#### 2.3.4. Discussion on Structure-Activity Relationship of APs

APs derived from dietary foods have given rise to more attention because they show enormous potential to be applied for both food and medical purposes due to their capability to promote human health by reducing oxidative stress [2,4]. Clarifying the structure-–function relationship will contribute to predicting the bioactivities of peptides and screening the high-quality protein materials for the preparation of APs [7,22]. However, the antioxidant mechanism of food-derived peptides have not been fully worked out. Yet, investigations by Sila and Bougatef [2] and Pan et al. [5] have indicated that the biological functions of peptides are distinctly affected by their amino acid composition, amino acid sequence, and molecular size. In addition, spatial conformation is thought to play a key role in their biological activities [31,32].

APs can act as lipid peroxyl radical trap and hydrogen donor to play their functions [1]. Short peptides containing 2 to 10 amino acids usually show better activity than their parent native proteins do because they can easily react with active radicals, which allows them to display their potent actions in reaction system [2,33]. In the experiment, F7-P3, F7-P5 and F7-P8 showed high antioxidant effect in the assays of radical scavenging, reducing power, and lipid peroxidation inhibition. These findings demonstrated that F7-P3 with five amino acid residues, F7-P5 with six amino acid residues, and F7-P8 with four amino acid residues could more easily and effectively react with free radicals and inhibit the propagating systems of lipid peroxidation in the tested model. Nevertheless, the antioxidant activity of F7-P1 (tripeptide) with the smallest molecular size among eight isolated peptides, was lower than those of F7-P3, F7-P5, and F7-P8. In addition, the reducing power of F7-P8 was weaker than those of F7-P3 and F7-P5. These findings imply that the bioactivities of APs were not only independent of molecular size, but also relied on other structural properties, such as their amino acid composition and amino acid sequences.

It has been proven that the amino acid composition and sequence distinctively influence the bioactivities of APs. Previous investigations showed that Glycine (Gly) residue could supply high backbone flexibility of peptides and neutralize active radical species through donating the single hydrogen atom of its side chain [1,34]. Gly is the richest amino acid in the sequences of collagens, and all members belonging to the collagen family are manifested by repetition domains of the tripeptides (Gly-X-Y) and drawn into the structure of the triple helix [18]. As showed in Table 1, eight isolated collagen peptides (F7-P1 to F7-P8) from hydrolysate of Spanish mackerel skins had the number of Gly residues ranging from one to three, which could be contributed to their antioxidant activities.

Zhao et al. [1] and Gimenez et al. [8] reported that polar amino acids, such as acidic (Asp and Glu) and basic (Lys, Arg, and His) amino acid residues in peptide sequences have a critical function in the HO• scavenging activities through metal ion chelating, which is positively related to the amino and carboxyl groups of their side chains. Arg and His residues in FPYLRH, and Glu residue in GIEWA show a significant effect on their oxidation resistance [1]. Glu, Asp, and Arg residues in LDEPDPLI and NTDGSTDYGILQINSR showed significant impact on their radical scavenging capacity [35]. Therefore, Asp residues may have a positive influence on the antioxidant capacity of F7-P3.

Hydrophobic amino acids in APs have their significant radical scavenging ability because their non-polar aliphatic groups have been shown to have strong reaction activity to hydrophobic PUFAs [1,2,3]. Aromatic amino acids (Phe and Tyr residues) can maintain ROS at a stable state by donating protons to free radicals that are deficient in electrons [6,32]. Pyrrolidine ring of Pro residue can strengthen the backbone flexibility of bioactive peptides and remove singlet oxygen because of its property of low ionization potential [5]. Zhao et al. [1] proved that Tyr residues could interdict the chain reaction of peroxidation mediated by free radicals because it could shift and change free radicals into steady phenoxy radicals. As a consequence, the hydrophobic and aromatic amino acid residues in the sequences of F7-P3 (Pro and Phe residues), F7-P5 (Pro, Tyr, and Lys residues), and F7-P8 (Tyr, Pro, and Met residues) should have an active influence on their activities of lipid peroxidation inhibitory and radical scavenging. Hydroxylproline (Hyp) is an important component in collagens, but no Hyp was found in the amino acid sequences of eight collagen peptides (F7-P1 to F7-P8). The reason for this phenomenon needs careful study in the future.

Therefore, the composition of amino acids and molecular size of eight collagen peptides (F7-P1 to F7-P8) plays a key role in their antioxidant activities. Small molecular size and hydrophobic amino acids help them access free radicals in an oxidant/antioxidant system with increased ease and efficiency. Moreover, antioxidant amino acids further change free radicals into more stable structures or inhibit the propagation of the peroxidizing chain reaction mediated by free radicals.

## 3. Experimental Section

### 3.1. Materials

Spanish mackerel (*S. niphonius*) were kindly supplied by Zhejiang Hailisheng Group Co., Ltd. (Zhejiang, China) and authenticated by Professor Sheng-long Zhao (Zhejiang Ocean University, Zhoushan, China). The skins of Spanish mackerel were corrected from the processing workshop of Zhejiang Hailisheng Group Co., Ltd. and stored at −20 °C. DPPH, Superdex^®^ Peptide 10/300 GL column, and ABTS were bought from Sigma-Aldrich (Shanghai) Trading Co., Ltd. (China). Trifluoroacetic acid (TFA) and Acetonitrile (ACN) of chromatographic grade were purchased from Thermo Fisher Scientific Co., Ltd. (Shanghai, China). Eight peptides (purity > 98%) including GPY, GPTGE, PFGPD, GPTGAKG, PYGAKG, GATGPQG, GPFGPM, and YGPM were synthesize by China Peptides Co., Ltd. (Suzhou, China).

### 3.2. Isolation of APs from Hydrolysate Fraction of F7

The preparation processes of collagens, collagen hydrolysate, and hydrolysate fraction (F1 to F7) from Spanish mackerel skins were performed following a previously established protocol [18,19]. F7 solution (500 μL, 25.0 mg/mL) was further purified by ÄKTA avant 25 (GE Healthcare Life Sciences, Chicago, IL, USA) in a Superdex^®^ Peptide 10/300 GL column. The flow rate of mobile phase (Deionized water (DW)) was 0.75 mL/min and monitored at 214 nm. According to the chromatographic peaks, four fractions, defined as FSP-I, FSP-II, FSP-III, and FSP-IV, were collected and freeze-dried. Finally, FSP-III (100 μL, 25.0 mg/mL) with higher HO• scavenging activity than the other three fractions was purified on a Kromasil C18 column (10 × 250 mm) in RP-HPLC system. The sample was eluted at a flow rate of 0.8 mL/min by a linear gradient of ACN (0% to 40% for 0 to 25 min) in 0.05% TFA. The absorbance of the eluate was monitored at 214 nm. According to the chromatographic peaks, eight peptides (F7-P1 to F7-P8) were collected and freeze-dried.

### 3.3. Analysis of Antioxidant Activity

#### 3.3.1. Radical Scavenging Activity

The scavenging activities of eight isolated peptides (F7-P1 to F7-P8) on HO•, DPPH•, $O_{2}^{-}$•, and ABTS^+^• were measured according to previous methods [20,36]. The results were expressed as a half elimination ratio (EC_50_) defined as the concentration where a sample caused a 50% decrease of the initial radical concentration. The counting method was based on the linear relationship between radical scavenging rates and peptide concentrations [1].

##### DPPH• Scavenging Activity

Two millilitres of samples consisting of distilled water (DW) and different concentrations of the analytes were placed in cuvettes, and 500 μL of an ethanolic solution of DPPH (0.02%) and 1.0 mL of ethanol were added. A control sample containing the DPPH solution without the sample was also prepared. In the blank, the DPPH solution was substituted with ethanol. The antioxidant activity of the sample was evaluated using the inhibition percentage of the DPPH• with the following equation:

DPPH• scavenging activity (%) = (A_0_ + A′ − A)/A_0_ × 100%,

where A is the absorbance rate of the sample, A_0_ is the control group absorbance, and A’ is the blank absorbance.

##### HO• Scavenging Activity

1.0 mL of a 1.87 mM 1,10-phenanthroline solution and 2.0 mL of the sample were added to a screw-capped tube and mixed. Following this, 1.0 mL of a FeSO_4_·7H_2_O solution (1.87 mM) was added to the mixture. The reaction was initiated by adding 1.0 mL of H_2_O_2_ (0.03%, v/v). After incubating at 37 °C for 1.0 h in a water bath, the absorbance of the reaction mixture was measured at 536 nm against a reagent blank. The reaction mixture, without any antioxidant, was used as the negative control, and a mixture without H_2_O_2_ was used as the blank. The HO• scavenging activity (HRSA) was calculated using the following formula:

HRSA (%) = [(A_s_ − A_n_)/(A_b_ − A_n_)] × 100%,

where A_s_, A_n_, and A_b_ are the absorbance values determined at 536 nm of the sample, the negative control, and the blank after the reaction, respectively.

##### $O_{2}^{-}$• Scavenging Activity

Superoxide anions were generated in 1 mL of nitrotetrazolium blue chloride (NBT) (2.52 mM), 1 mL of NADH (624 mM), and 1 mL of different sample concentrations. The reaction was initiated by adding 1 mL of phenazine methosulphate solution (120 μM) to the reaction mixture. The absorbance was measured at 560 nm against the corresponding blank after 5-min incubation at 25 °C. The scavenging capacity of the $O_{2}^{-}$• was calculated using the following equation:

$$O_{2}^{-}•\;\mathrm{scavenging}\;\mathrm{activity}\;(\%)=[(A_{\mathrm{control}}-A_{\mathrm{sample}})/A_{\mathrm{control}}]\times 100\%,$$

where A_control_ is the absorbance without sample and A_sample_ is the absorbance with sample.

##### ABTS^+^• Scavenging Activity

ABTS^+^• was generated by mixing an ABTS stock solution (7 mM) with K_2_S_2_O_8_ (2.45 mM). The mixture was left in the dark for 16 h at room temperature. The ABTS^+^• solution was diluted in 5 mM phosphate buffered saline (PBS, pH 7.4) to an absorbance of 0.70 ± 0.02 at 734 nm. One millilitre of diluted ABTS^+^• solution was mixed with one millilitre of the different sample concentrations. After 10 min, the absorbances at 734 nm were measured against the corresponding blank. The ABTS^+^• scavenging activities of the samples were calculated using the same equation as indicated in $O_{2}^{-}$• scavenging activity (%).

#### 3.3.2. Reducing Power Assay

The reducing power assay of eight isolated peptides (F7-P1 to F7-P8) was carried out according to previously reported methods [21,37]. We blended 2.5 mL of 1% K_3_Fe(CN)_6_ solution with 2.0 mL of peptide solution and incubated at 50 °C for 30 min. After that, 1.5 mL of 10% trichloroacetic acid was added into the mixed solution. Finally, 2.0 mL of the upper layer, 0.5 mL of 0.1% aqueous FeCl_3_, and 2.0 mL of DW were mixed, and the absorbance at 700 nm was applied to record the reaction mixture.

#### 3.3.3. Lipid Peroxidation Inhibition Assay

This assay was performed according to previously reported methods [20,38]. In brief, 5.0 mg of a sample dissolved in 10 mL of phosphate buffer saline (50 mM, pH 7.0) was mixed with 0.13 mL of linoleic acid solution and 10 mL of absolute ethyl alcohol. The total volume was added to 25 mL with DW in a conical flask. In a dark room, the mixed solution was kept warm at 40 °C. Subsequently, 100 μL of the solution was blended with 0.1 mL of 30% NH_4_CNS, 4.7 mL of 75% ethanol, and 0.1 mL of 20 mM FeCl_2_ in 3.5% HCl. After 3 min, the Fe(SCN)_3_ value was recorded at 500 nm following color development with thiocyanate and FeCl_2_ every 24 h during the incubation period at 40 °C.

### 3.4. Determination of Molecular Mass and AA Sequences

Molecular masses and AA sequences of F7-P1 to F7-P8 were measured according to previously reported methods [19]. A Q-TOF mass spectrometer (Micromass, Waters, Milford, MA, USA) combined with an electrospray ionization (ESI) were used to determine the molecular masses of F7-P1 to F7-P8. N-terminal AA sequencing of F7-P1 to F7-P8 were measured on an Applied Biosystems 494 protein sequencer (Perkin Elmer/Applied Biosystems Inc., Foster City, CA, USA). 

### 3.5. Statistical Analysis

Data are presented as means ± standard deviation (SD) (*n* = 3). ANOVA test (SPSS 19.0 software) was used to comparative analysis the mean value of each treatment. Duncan′s multiple range test was carried out to analyze the significant differences of samples (*p* < 0.05). 

## 4. Conclusions

In this study, eight collagen peptides (F7-P1 to F7-P8) from protein hydrolysate fraction (F7) of Spanish mackerel skins were isolated and identified as GPY, GPTGE, PFGPD, GPTGAKG, PYGAKG, GATGPQG, GPFGPM, and YGPM. Among them, PFGPD, PYGAKG, and YGPM could effectively inhibit lipid peroxidation and scavenge free radicals in a dose–effect relationship. The present results indicate that eight isolated collagen peptides (F7-P1 to F7-P8) are good candidates as antioxidant additives for various industrial applications. Nonetheless, it is essential to carry out further investigations on the antioxidant mechanism and relationship between the structure and activity of the eight isolated collagen peptides using cell and animal models.

## Figures and Tables

**Table 1:** Retention time (RT), amino acid (AA) sequences, and molecular masses of eight antioxidant peptides (F7-P1 to F7-P8) from collagen hydrolysate fraction of F7 of Spanish mackerel (*S. niphonius*) skin.

	RT (min)	AA Sequence	Theoretical Mass/Observed Mass (Da)
F7-P1	4.951	GPY	335.36/335.31
F7-P2	8.561	GPTGE	459.45/459.47
F7-P3	9.704	PFGPD	531.56/531.52
F7-P4	10.245	GPTGAKG	586.64/586.65
F7-P5	10.804	PYGAKG	591.66/591.69
F7-P6	11.892	GATGPQG	586.60/586.61
F7-P7	13.389	GPFGPM	604.72/604.73
F7-P8	13.612	YGPM	466.55/466.50

**Table 2:** EC_50_ values of eight antioxidant peptides (F7-P1 to F7-P8) from collagen hydrolysate fraction (F7) of Spanish mackerel (*S. niphonius*) skin on four kinds of free radicals.

	EC_50_ (mg/mL)			
	DPPH•	HO•	$O_{2}^{-}$•	ABTS^+^•
F7-P1	1.01 ± 0.09 ^a^	3.22 ± 0.21 ^a^	3.98 ± 0.26 ^a^	2.12 ± 0.16 ^a^
F7-P2	1.46 ± 0.12 ^b^	2.13 ± 0.15 ^b^	2.96 ± 0.19 ^b^	4.51 ± 0.24 ^b^
F7-P3	0.80 ± 0.09 ^a^	0.81 ± 0.05 ^c^	0.91 ± 0.08 ^c^	0.86 ± 0.05 ^c^
F7-P4	4.63 ± 0.21 ^c^	2.27 ± 0.14 ^b^	1.73 ± 0.11 ^d^	3.27 ± 0.27 ^d^
F7-P5	3.02 ± 0.19 ^d^	0.66 ± 0.08 ^c^	0.80 ± 0.06 ^c^	1.07 ± 0.10 ^c^
F7-P6	3.65 ± 0.30 ^e^	2.11 ± 0.16 ^b^	7.21 ± 0.27 ^e^	3.86 ± 0.19 ^e^
F7-P7	7.93 ± 0.52 ^f^	5.24 ± 0.32 ^d^	9.83 ± 0.35 ^f^	8.54 ± 0.43 ^f^
F7-P8	0.72 ± 0.06 ^a^	0.88 ± 0.09 ^c^	0.73 ± 0.06 ^c^	0.82 ± 0.04 ^c^

All data are presented as the mean ± SD of triplicate results. ^a–f^ Values with same letters indicate no significant difference of different sample at same radicals (*p* > 0.05).
